# Zero-TE MRI-based attenuation correction for bone components on chest [^18^F] FDG PET/MRI: accuracy, repeatability, and external validation of an unsupervised deep learning approach using unpaired PET/CT data

**DOI:** 10.1007/s12149-026-02213-0

**Published:** 2026-05-09

**Authors:** Munenobu Nogami, Hidetoshi Matsuo, Mizuho Nishio, Feibi Zeng, Junko Inoue Inukai, Miho Tachibana, Florian Wiesinger, Sandeep Kaushik, Takako Kurimoto, Kazuhiro Kubo, Martin W. Huellner, Hidehiko Okazawa, Takamichi Murakami

**Affiliations:** 1 Department of Radiology, Kobe University Graduate School of Medicine, 7- 5-2 Kusunoki-cho, Chuo-ku, Kobe, 650-0017 Hyogo Japan; 2 Division of Medical Imaging, Biomedical Imaging Research Center, University of Fukui, Fukui, Japan; 3 GE HealthCare, Munich, Germany; 4 GE HealthCare, Hino, Japan; 5 Department of Nuclear Medicine, University Hospital Zurich, University of Zurich, Zurich, Switzerland

**Keywords:** PET/MRI, attenuation correction, zero echo time, deep learning, chest

## Abstract

**Objective:**

In positron emission tomography (PET)/magnetic resonance imaging (MRI), attenuation correction (AC) for PET of the head is achieved by MRI data to generate pseudo-computed tomography (CT) images. However, for the torso, AC becomes more challenging due to the complexity of separating bone components. Additionally, generating accurate MRI-based CT using deep learning poses significant difficulties for the chest, primarily because perfectly paired MRI and CT training data are hard to obtain owing to respiratory motion and body movements. We previously demonstrated that MRI-to-CT conversion can be achieved without deformation, even using unsupervised learning for zero echo time (ZTE) MRI and CT data from different individuals. Building on this foundation, our study aims to apply this approach to AC in chest PET/MRI and assess their quantitative accuracy, reproducibility, and external validity.

**Methods:**

The datasets used included (1) training dataset (unpaired ZTE MRI and CT of PET/CT, *n* = 360 and 500, respectively); (2) test dataset (paired PET/MRI and PET/CT, *n* = 25 and 25, respectively); (3) repeatability assessment dataset (repeated PET/MRI, *n* = 15 × 2 scans for the same patient); and (4) external validation dataset (paired MRI component of PET/MRI and CT, *n* = 30 and 30, respectively, acquired at another institution). Unpaired training data were used to train the deep learning model of pseudo-CT generation from ZTE. The accuracy, repeatability, and reproducibility of the PET/MRI scans using ZTE- and deep learning-based AC (MRAC_ZTE_) were evaluated based on the similarity of the histograms and the mean standardized uptake value (SUVmean) of physiological background of bone and liver.

**Results:**

The histogram correlation coefficients between MRAC_ZTE_ and the AC map based on the CT (CTAC) for the spine were significantly higher than those between conventional AC (MRAC_Dixon_) and CTAC. Additionally, bone SUVmean obtained using MRAC_ZTE_ showed reduced bias relative to CTAC compared with MRAC_Dixon_. This method proved to be reproducible on each patient level and robust against external validation.

**Conclusions:**

Unsupervised learning with unpaired ZTE and CT data enabled pseudo-CT generation with bone components that closely matched CT-based attenuation maps. Integration into MR-based attenuation correction resulted in stable physiological uptake measurements in chest PET/MRI, supporting the feasibility of this approach.

**Supplementary Information:**

The online version contains supplementary material available at 10.1007/s12149-026-02213-0.

## Introduction

 The lack of computed tomography (CT) capability in integrated positron emission tomography/magnetic resonance imaging (PET/MRI) systems represents a significant limitation compared to PET/CT systems, as it makes PET attenuation correction (AC) relatively more challenging. Generating gamma-ray AC maps using pseudo-CT derived from MRI data (i.e., MR-based attenuation correction, [MRAC]) is a commonly used method [1]. However, conventional MRI techniques, such as the T1-weighted two-point Dixon method, struggle to accurately depict bone components. These methods typically assign fixed attenuation coefficient (mu) values at 511 keV for air, lung, fat, and soft tissue attenuation (0, 0.018, 0.086, 0.1 [cm^− 1^], respectively) while neglecting bone attenuation [2, 3]. This leads to an underestimation of radiotracer uptake in or near bone structures and regions surrounded by bone compared to PET/CT [4], thereby diminishing the inherent advantages of PET/MRI.

MRI sequences with very short echo times, such as the zero-echo time (ZTE) method, provide excellent bone and lung delineation [5]. Additionally, the signal intensity in ZTE images measured in arbitrary units is known to inversely correlate with the X-ray attenuation coefficient (i.e., Hounsfield Unit, HU) of bone in CT [6]. This correlation makes ZTE an effective technique for deriving bone ACs in PET/MRI. Notably, ZTE has already been successfully employed for AC with a bone component in head imaging [7–9].

In the trunk, however, generating the bone component of CT remains difficult, even with ZTE, due to the presence of MRI signals similar to those of bone, such as those from the lungs and intestinal gas [10]. To address this limitation, deep learning methods for MRI-to-CT conversion have been employed. However, obtaining paired MRI and CT datasets for training [11] is inherently challenging as MRI and CT images of the torso are typically acquired using different scanners, leading to discrepancies in body positioning and respiratory states between scans. Tri-modality systems might represent a solution but are no longer on the market [12, 13]. Obtaining high-quality paired training data is particularly challenging for highly deformable organs, such as those in the chest. Thus, unsupervised learning becomes essential.

**Fig. 1:**
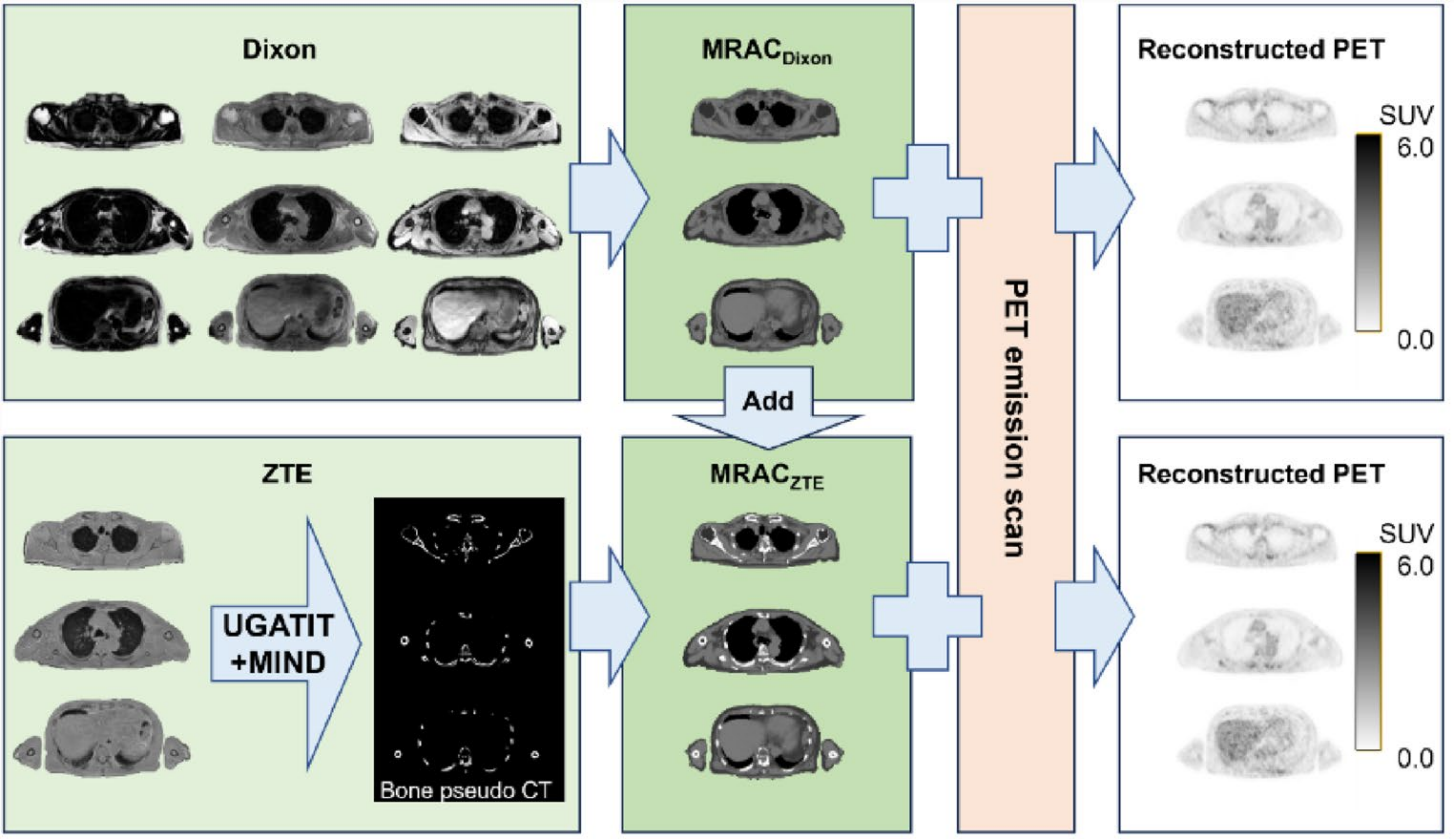
Diagrammatic representation of the process of PET image reconstruction using the AC map based on the conventional method (MRAC_Dixon_) and the AC map utilizing deep learning and ZTE MRI (MRAC_ZTE_). AC, attenuation correction; ZTE, zero echo-time

In a previous study, we successfully generated the bone component of CT with continuous HU values using ZTE by training with unpaired ZTE and CT datasets for the chest [14]. This unsupervised learning was achieved by incorporating a common image identifier (modality independent neighborhood descriptor [MIND]) [15] into the loss function of unsupervised generative adversarial networks with adaptive layer-instance normalization for image-to-image translation (U-GAT-IT) [16]. Using this cycle generative adversarial network (cGAN) framework, MRI-to-CT conversion was performed through unsupervised learning of unpaired ZTE and CT datasets (Figure. 1). Despite these advancements, further evaluation is needed to assess the quantitative accuracy, intra-patient repeatability, and robustness against external datasets of this method for PET reconstruction before clinical applications.

While our previous work established the fundamental feasibility of generating pseudo-CT from ZTE-MRI using unsupervised learning, the present study further extends this approach to clinical PET/MRI quantification. This work focuses on integrating the generated bone maps into the attenuation correction pipeline and systematically evaluating its impact on SUV accuracy in the chest, which was not addressed in our prior technical report. We hypothesized that U-GAT-IT combined with MIND could accurately generate the bone component of CT from ZTE data acquired with a PET/MRI system, enabling effective AC for PET imaging in the chest region.

Our study aimed to evaluate the quantitative accuracy, repeatability, and robustness of chest PET/MRI performed using AC based on the bone component of CT derived from ZTE data via a deep learning model employing unpaired and unsupervised learning.

## Materials and methods

### Patients and datasets

This retrospective study was approved by the institutional review boards (institutional ethics committee approval no. B210206 in Institute 1 and no. 20220184 in Institute 2). Given its retrospective design, the requirement for written informed consent was waived.

**Fig. 2:**
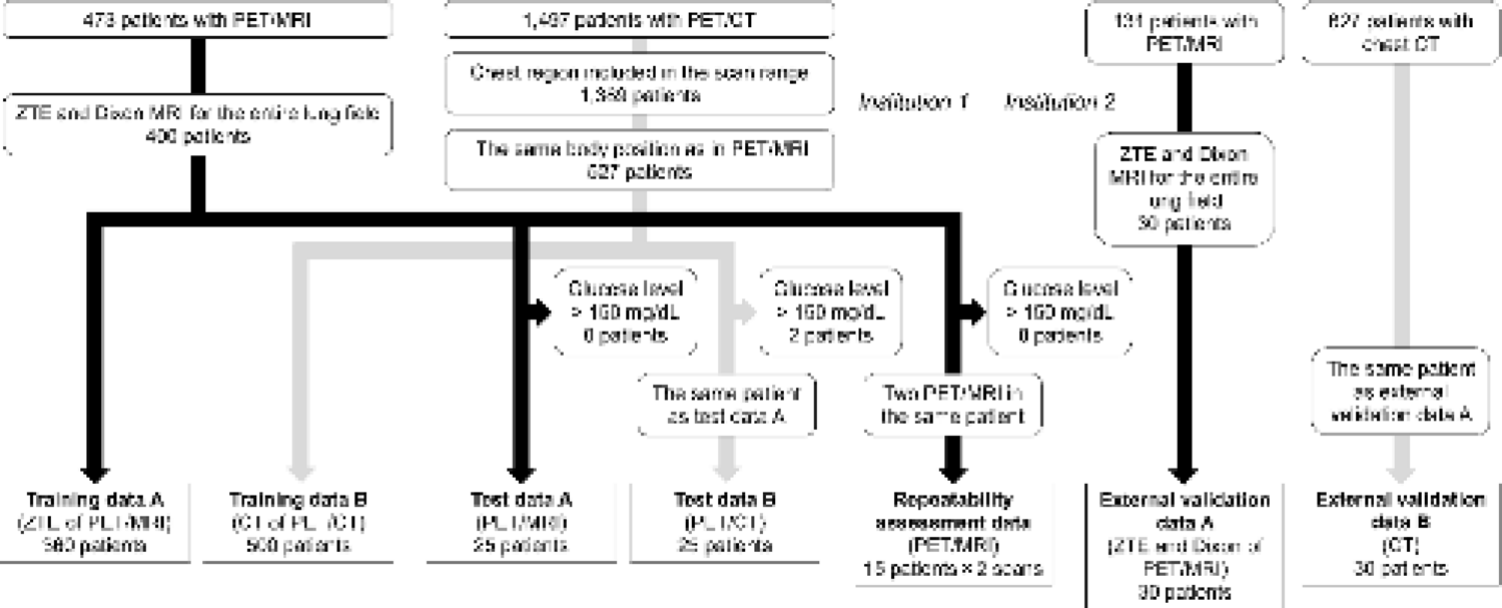
Inclusion and exclusion criteria for the study. All datasets were mutually exclusive at the patient level. ZTE, zero echo-time

Seven datasets were used in this study: (1) training dataset A (ZTE MRI), (2) training dataset B (CT of PET/CT), (3) test dataset A (PET/MRI), (4) test dataset B (PET/CT), (5) repeatability assessment dataset (PET/MRI), (6) external validation dataset A (MRI components of PET/MRI), and (7) external validation dataset B (CT). Consecutive [^18^F] FDG PET/MRI and [^18^F] FDG PET/CT examinations (473 and 1,497, respectively) performed at institute 1 were retrospectively analyzed. Consecutive [^18^F] FDG PET/MRI and CT examinations (131 and 627, respectively) performed at institute 2 were also retrospectively evaluated. The inclusion criterion and exclusion criteria are shown in Fig. 2. PET/MRI scans at institution 1 and 2 were performed according to the same scan protocol.

A total of 360 ZTE MRI and 500 CT components from PET/CT were used as training datasets A and B, respectively, for training the deep learning model. Due to the difference in slice thickness between training datasets A and B, the number of cases in B was set higher than in A to match the total number of slices as closely as possible. To test the model, 25 PET/MRI and PET/CT scans from the same patients were used as test datasets A and B. Two patients with PET/CT scans were excluded from the test dataset B due to blood glucose levels > 150 mg/dL. Repeatability was assessed using 15 test-retest PET/MRI scans of the same patients. For external validation, 30 ZTE MRI scans from PET/MRI and 30 CT scans from institution 2 were included.　To prevent data leakage, strict patient-level separation was enforced. No patient contributing to the training datasets (ZTE or CT) was included in any test, repeatability, or external validation datasets. In this study, external validation was defined as assessment of pseudo-CT generation and attenuation-map consistency across institutions. Because CT data in the external cohort were acquired independently and were not associated with PET/CT examinations, PET-based quantitative validation was not feasible in this dataset. Patient demographics for each dataset are provided in Supplemental Table 4.

### PET/MRI

The PET and MRI parameters used in our study are standardized and summarized in Supplemental Tables 1 and 2. All patients fasted for at least 6 h before the examinations and were administered 3.5 MBq/kg of [^18^F] FDG. An integrated 3.0 T PET/MRI scanner (SIGNA PET/MR, GE HealthCare, Chicago, Illinois, USA) was used for all examinations.

For chest PET/MRI, a PET emission scan and both Dixon and ZTE MRI scans were acquired simultaneously in the same bed position. ZTE MRI was performed under free breathing using three-dimensional (3D) center-out radial sampling and a TE of zero, providing isotropic resolution at 2 mm^3^ with a field of view of 50 cm; the approximate acquisition time was 5 min. To mitigate fat-water chemical shift effects at fat-water tissue boundaries, a high imaging bandwidth (± 62.5 kHz) was used. Additionally, the imaging center frequency was carefully adjusted to lie between those of fat and water, resulting in in-phase contrast ZTE images with uniform soft-tissue signal intensity and minimal interference from fat-water interactions [17].

For the repeatability analysis, the two PET/MRI scans were performed within 35 days of each other (mean interval, 32.2 ± 2.0 days), with no intervening treatment or other clinical intervention, using the same acquisition protocol with matched injected activity (3.5 MBq/kg), uptake time (59.8 ± 5.6 min), and scan duration (2.5 min) (Supplemental Table 1).

### Image post-processing

ZTE image processing was performed using a semi-automated background signal removal technique, involving thresholding and filling-in method on the commercially available Advantage Workstation v4.7 (GE HealthCare). To address sensitivity variations and standardize the ZTE images to the median tissue value, the nonparametric N4ITK method was used [18]. Concurrently, the workstation was used to modify the CT images by removing scanner beds. For data normalization, a window width and window level of 800 and 600 for ZTE, and 2000 and 350 for CT were specified. The ZTE and CT images were then downscaled to a resolution of 256 × 256 pixels for subsequent model training due to the limited volume of data that could be processed by the graphics processing unit on the workstation.

### Deep learning

The deep learning method used in this study (Supplemental Fig. 1) has been described in detail elsewhere [14]. Briefly, the algorithm is based on U-GAT-IT [16], an encoder–decoder method for image generation that incorporates an attention module in both the discriminator and generator, along with adaptive layer-instance normalization (AdaLIN) to focus on relevant parts of the image. The MIND algorithm [15] involves multi-modal deformable registration to extract numerical descriptors preserved across modalities by capturing local feature structures (Supplemental Fig. 2). This was incorporated as a loss function in U-GAT-IT to minimize anatomical structure changes and eliminate distortions in the generated images, even when training with unpaired ZTE and CT data. The MIND-based loss used in this study was originally introduced in our previous work [14] for unpaired ZTE–CT translation.

In the present study, a 2.5D pseudo-color input strategy was additionally implemented to improve structural continuity in the through-plane direction. U-GAT-IT was initially designed to process three-channel RGB images, but the CT and MRI images used in our study were grayscale, single-channel images. Therefore, to incorporate simple 3D information, we first created pseudo-color images by placing the target slice image in the central channel and the slices immediately before and after it in the remaining two channels. These pseudo-color images were then used as inputs for the model during training. For the final generation of pseudo-CT images, only the central channel of a generated pseudo-color image was extracted and adopted as the output. The 2.5D pseudo-color strategy was introduced as pragmatic design choices to improve structural continuity and cross-modality consistency in an unpaired learning setting(Supplemental Figs. 3–4 and Supplemental Table 5). These components were adopted as implementation choices within the existing framework. The present study was not designed to isolate the contributions of the 2.5D input to PET quantitative performance. All processing was performed on a commercially available Graphics Processing Unit (NVIDIA RTX A5000, 24 GB)-equipped workstation (Linux, Intel Xeon E5-1650 v4, 3.6 GHz, 32 GB).

### PET reconstruction

**Fig. 3:**
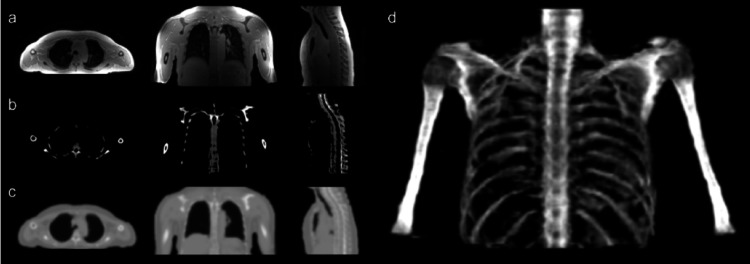
Pseudo-CT generation of bone components from ZTE MRI using unpaired and unsupervised deep learning. Examples of ZTE for the chest (**a**), pseudo-CT of the bone component (**b**, **d**) and attenuation correction (mu) map with bone component (**c**). CT, computed tomography; MRI, magnetic resonance imaging; ZTE, zero echo-time

Two MRAC maps were generated for assessment: a 4-class AC map (MRAC_Dixon_), i.e., a conventional Dixon MRI-based AC map with four tissue (air, lung, fat, and soft tissue) segmentation and fixed mu value adaptation, and a 5-class AC map (MRAC_ZTE_) including bone tissue, i.e., a hybrid AC map with Dixon and ZTE sequences obtained by superimposing the bone component of pseudo-CT with continuous HU values generated by deep learning from ZTE on the MRAC_Dixon_ map (Fig. 3). The continuous attenuation coefficients of bone were calculated by converting the continuous HU values using a standard conversion formula widely used in PET/CT [3]. The implementation details of the hybrid AC map is shown in Supplemental Fig. 5. PET reconstruction was performed with an emission scan duration of 2.5 min using time of flight ordered subset expectation maximization. All corrections, including scatter correction, were performed using the standard method of the scanner without modification. All PET reconstructions for PET/MRI in our study were performed on an offline workstation using a MATLAB PET reconstruction software (Duetto v02.18, GE HealthCare).

### Image analysis

Linear attenuation coefficients (mu) are reported at 511 keV in units of cm^− 1^. HU values were converted to mu values using a bilinear transformation.

To assess the differences in mu value at 511 keV of tissues on the AC maps, 1.5 cm cubic and fixed volumes of interest (VOIs) were manually placed on subcutaneous fat, lung, soft tissue (the erector spinae muscle), and bone (the 3rd, 7th, and 11th thoracic vertebrae and the posterior aspect of the right 7th rib) for MRAC_Dixon_, MRAC_ZTE_, and the AC map based on the CT component of PET/CT (CTAC). The assessed areas of the interests were selected as a representative and relatively stable anatomical landmark in the thoracic region, as it is less affected by respiratory excursion and rib curvature compared with other thoracic structures.

To compare the histogram of bone mu value at 511 keV on AC maps, 3.0 cm cubic and fixed VOIs on the bone in MRAC_Dixon_, MRAC_ZTE_ and CTAC were evaluated (Supplemental Fig. 6).

To assess the semi-quantitative value of physiological [^18^F] FDG uptake in reconstructed PET using the respective AC map, 1.5 cm cubic VOIs were placed on the bone and liver in each PET reconstruction by MRAC_Dixon_, MRAC_ZTE_ and CTAC. The average standardized uptake value (SUV) in the VOIs, normalized to the patient’s body weight (SUVmean), was calculated as a semi-quantitative measure of PET.

### Reference standard

As a reference standard for the AC map and reconstructed PET, the CT and PET of PET/CT data for the same patient was defined as test dataset B. An integrated PET/CT scanner (Discovery PET/CT 690, GE HealthCare, Chicago, Illinois, USA) was used for the patients in test dataset B. CT and PET reconstruction parameters are shown in Supplemental Tables 1 and 3. In brief, PET image reconstruction was performed on the PET/CT system using manufacturer-recommended parameters. For external validation, CT data from the same patient were specified as external validation dataset B. Details regarding the CT protocol used in the external validation dataset B are provided in Supplemental Table 3.

### Statistical analysis

Bland–Altman plots were used to assess the mean differences and limits of agreement of HU values on MRAC and CTAC maps.

For the histogram comparison of each AC map, the correlation coefficients of MRAC_Dixon_ and MRAC_ZTE_ with CTAC were calculated for 20 bins of each histogram (H_CTAC_ and H_MRAC_) of a VOI according to the following formula:

$$\:d\left({H}_{CTAC},\:{H}_{MRAC}\right)=\frac{{\sum\:}_{I}({H}_{CTAC\left(I\right)}-{\stackrel{-}{H}}_{CTAC})({H}_{MRAC\left(I\right)}-{\stackrel{-}{H}}_{MRAC})}{\sqrt{{\sum\:}_{I}{({H}_{CTAC\left(I\right)}-{\stackrel{-}{H}}_{CTAC})}^{2}{\sum\:}_{I}{({H}_{MRAC\left(I\right)}-{\stackrel{-}{H}}_{MRAC})}^{2}}}$$

where $$\:{\stackrel{-}{H}}_{k}=\frac{1}{20}\sum\:_{J}H\_k\left(J\right)$$.

To compare the physiological bone and liver uptake of PET reconstruction using each AC map, SUVmeans for MRAC_Dixon_ and MRAC_ZTE_ were compared with those for CTAC, using Wilcoxon’s signed rank test. The mean differences and limits of agreement of bone SUVmeans were also evaluated using Bland–Altman plots.

To assess the reproducibility of the VOI measurements, intraclass correlation coefficient (ICC) of the measured HU values on pseudo-CT and actual CT between two readers were evaluated.

MedCalc^®^ version 22.018 (MedCalc Software Ltd., Ostend, Belgium; https://www.medcalc.org; 2024) was used for all statistical analyses. Statistical significance was set at *p* < 0.05.

## Results

Model training using U-GAT-IT+MIND was terminated after 9.5 × 10^5^ learning steps, at which point the loss functions stabilized at consistently low values (Supplemental Fig. 7).

For paired comparisons, values are reported as median (interquartile range, IQR). For Bland–Altman analyses, mean difference and limits of agreement are reported.

The ICCs of two readers for the mean HU values of volumes of interest (VOIs) placed in subcutaneous fat, lung, soft tissue, and bone for MRAC_Dixon_, MRAC_ZTE_, and CTAC were summarized in Supplemental Table 6. For all VOIs, the ICCs between the two readers were greater than 0.98. In particular, subcutaneous fat, lung, and soft tissue on MRAC_Dixon_ and MRAC_ZTE_, for which fixed mu-values are assigned, showed excellent interobserver agreement with ICCs exceeding 0.99; notably, the VOIs targeting bone components generated by deep learning also demonstrated high ICCs (> 0.98). Subsequent analyses were performed using the VOI measurements from Reader 1.

**Fig. 4:**
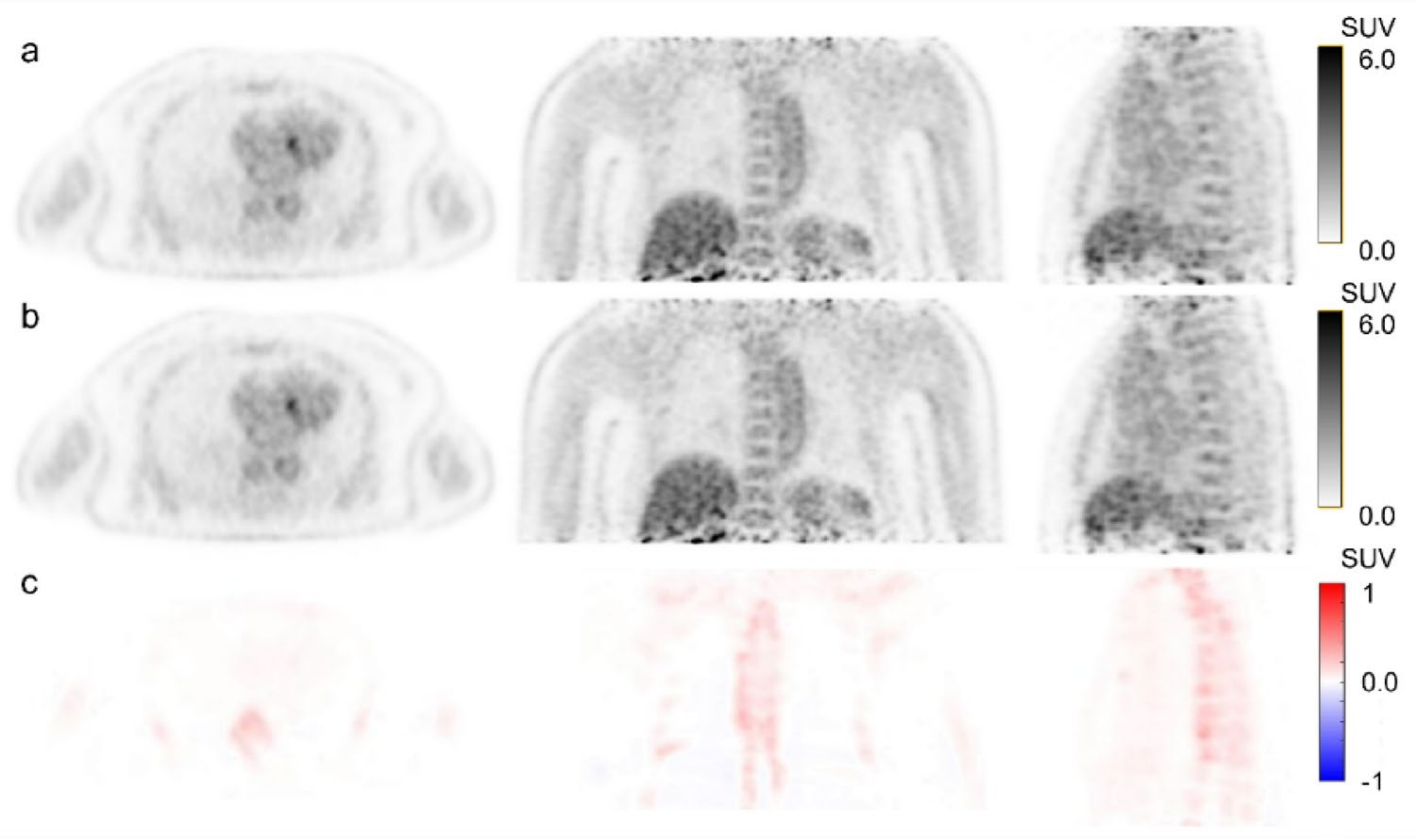
PET reconstructed by MRAC_Dixon_(**a**), MRAC_ZTE_(**b**) and SUV difference map (MRAC_ZTE_ − MRAC_Dixon_) (**c**). For this case, mean SUVmean within the 7th thoracic vertebral VOI was 1.07 for MRAC_ZTE_ and 0.96 for MRAC_Dixon_, corresponding to a difference of (MRAC_ZTE_ − MRAC_Dixon_) = 0.11 (11.5% relative increase). AC, attenuation correction; ZTE, zero echo-time

The differences in bone mu value at 511 keV between MRAC and CTAC for each tissue in the test dataset are shown in Table 1. The mean difference in bone mu value of MRAC_ZTE_ (-0.0041) were smaller than that of MRAC_Dixon_ (-0.0142). Figure 4 presents PET images with attenuation correction performed using MRAC_Dixon_ and MRAC_ZTE_, as well as the corresponding SUV difference map. Notable differences in SUV values are observed in bone, with MRAC_ZTE_–based attenuation correction resulting in higher SUVs compared with MRAC_Dixon_.

**Table 1:** Difference in bone mu values at 511 keV between the MRAC maps and the CT-based attenuation map (CTAC)

AC maps	Mean difference [cm^− 1^] (95% Confidence interval)	Upper limits of agreement [cm^− 1^] (95% Confidence interval)	Lower limits of agreement [cm^− 1^] (95% Confidence interval)
MRAC_Dixon_	-0.0142 (-0.0130 to -0.0154)	-0.0092 (-0.0118 to -0.0075)	-0.0186 (-0.0123 to -0.0168)
MRAC_ZTE_	-0.0041 (-0.0052 to – 0.0028)	0.0020 (-0.0003 to 0.0040)	-0.0099 (-0.0120 to -0.0073)

**Fig. 5:**
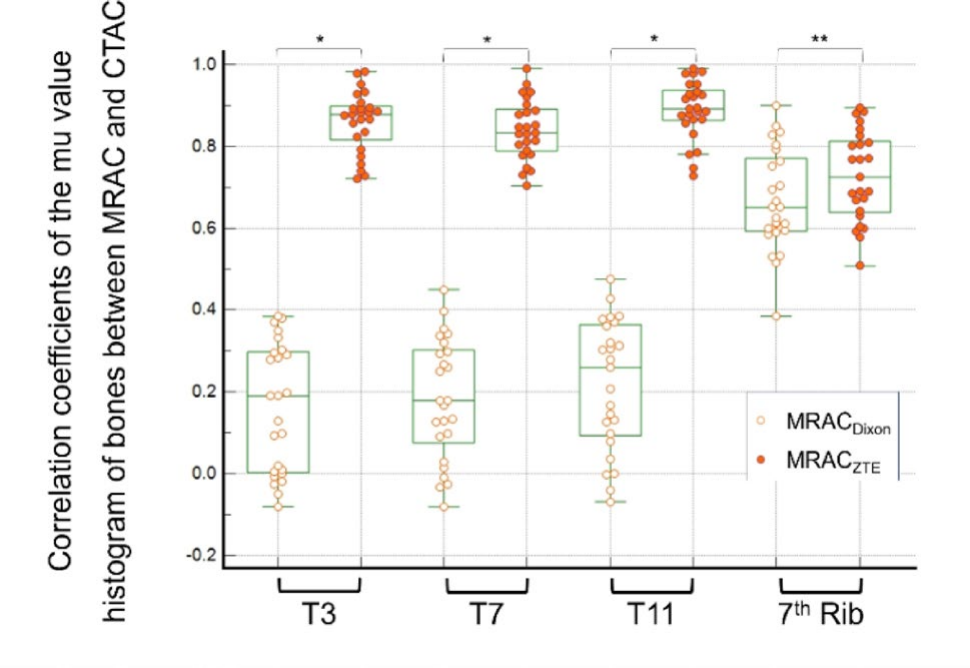
Box-and-whisker plots showing the correlation between actual CT and pseudo-CT in the bone mu values at 511 keV on the 3rd, 7th, and 11th thoracic vertebral body and 7th rib. *, *p* < 0.0001; **p < 0.005, MRAC, magnetic resonance attenuation correction; ZTE, zero echo-time; T, thoracic spine

The results of the correlation coefficient comparisons for histograms of bone mu value at 511 keV between MRAC and CTAC in the test dataset are shown in Fig. 5. The correlation coefficients for MRAC_ZTE_ and CTAC in 3rd thoracic vertebra (median, 0.88), 7th vertebra (median, 0.83), 11th vertebra (median, 0.89) and 7th rib (median, 0.72) were significantly higher than those for MRAC_Dixon_ and CTAC (0.19, 0.18, 0.26 and 0.65, respectively) (*p* < 0.005).

**Fig. 6:**
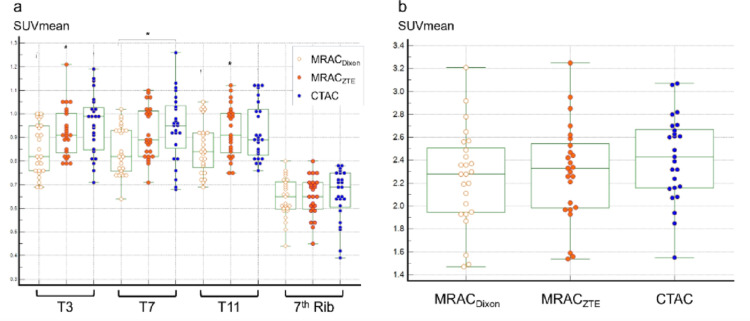
Box-and-whisker plots showing the differences in physiological uptake (SUVmean) for bone (3rd, 7th, and 11th thoracic vertebral body and 7th rib) (a) and liver (b) between PET reconstructed using actual CT and pseudo-CT. The mean SUV of the bone was significantly lower in PET using the 4-class MRAC (MRAC_Dixon_) compared to PET using actual CT (CTAC) (*, *p* < 0.05), while no significant difference was observed with the 5-class MRAC (MRAC_ZTE_). There were no significant differences in liver SUVmean. SUV, standardized uptake value; ZTE, zero echo-time; T, thoracic spine

The results of the SUVmean comparisons of physiological bone and liver on reconstructed PET between MRAC and CTAC in the test dataset are shown in Fig. 6. SUVmean of 3rd vertebra (median 0.82), 7th vertebra (median, 0.82), 11th vertebra (median, 0.84) on PET using MRAC_Dixon_ was significantly lower than that using CTAC (0.99, 0.95, 0.89, respectively) in the same patient (*p* < 0.05). Conversely, no statistically significant difference was observed between MRAC_ZTE_ and CTAC. There were no significant differences in 7th rib and liver SUVmean.

**Fig. 7:**
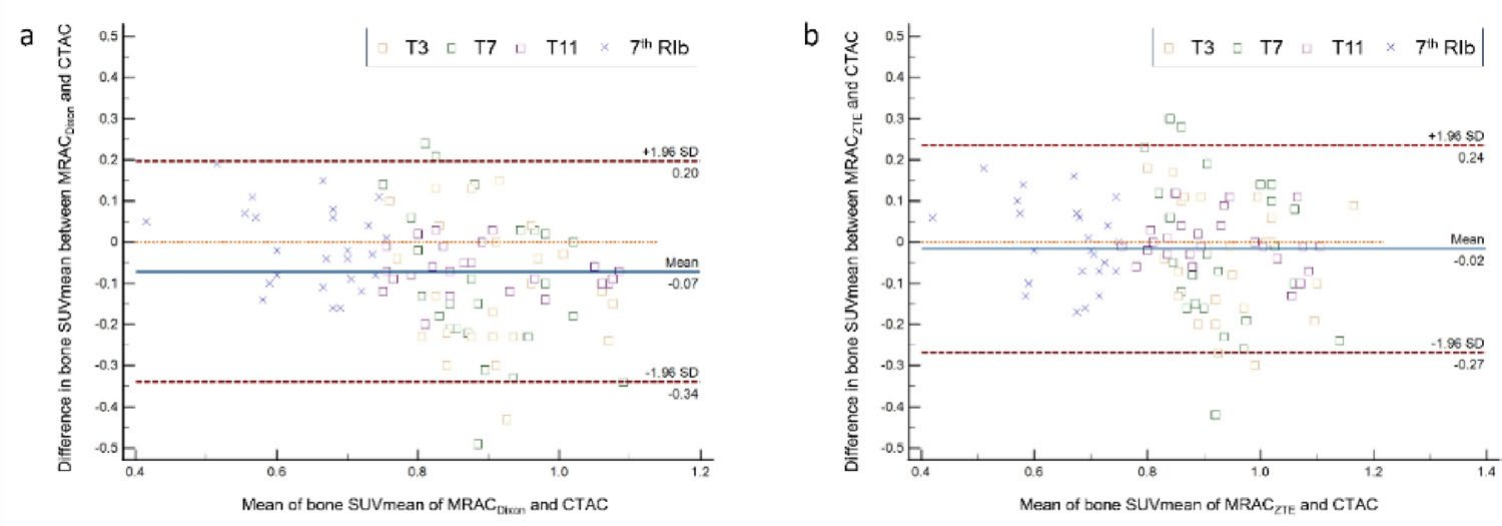
Bland–Altman plots showing the differences in bone SUVmean between MRAC and CTAC in the test data. The bone SUVmean has a smaller difference from the CTAC when using the 5-class MRAC (MRAC_ZTE_) (**b**), compared to the 4-class MRAC (MRAC_Dixon_) (**a**). SUV, standardized uptake value; ZTE, zero echo-time; T, thoracic spine

Differences in bone SUVmean between MRAC and CTAC in the test dataset are shown in Fig. 7. The mean difference between MRAC_Dixon_ and CTAC were − 0.07 (95% Confidence Interval [CI], -0.10 to -0.04) with upper and lower limits of agreement of 0.19 (CI, 0.15 to 0.24) and − 0.34 (CI, -0.38 to -0.29). Corresponding values for MRAC_ZTE_ and CTAC were − 0.02 (CI, -0.04 to 0.01) with 0.24 (CI, 0.19 to 0.28) and − 0.27 (CI, -0.31 to -0.23).

Table 2 presents the Bland–Altman plot for the repeatability assessment. The percent difference of bone SUVmean between two test-retest MRAC_ZTE_ were − 5.2%. Similarly, for liver SUVmean between two test-retest MRAC_ZTE_ scans, the values were − 5.4%.

**Table 2:** The difference in physiological uptake (SUVmean) for bone and liver using MRAC_ZTE_ in the repeatability assessment data (mean difference and limits of agreement)

Region	Mean difference (95% Confidence interval)	Upper limits of agreement (95% Confidence interval)	Lower limits of agreement (95% Confidence interval)	Unit
Bone	-0.08(-0.12 to 0.03) -5.21(-9.00. to 1.42)	0.26 (0.18 to 0.34) 22.78(16.26 to 29.29)	-0.41(-0.49 to -0.33) -33.21 (-39.72 to -26.69)	[%]
Liver	-0.08(-0.22 to -0.07) -5.35(-14.10 to 3.40)	0.45(0.19 to 0.70) 25.62(10.32 to 40.93)	-0.60(-0.85 to -0.34) -36.33(-51.63 to -21.02)	[%]

The results of the comparison of correlation coefficients of HU histograms of bone between MRAC and CT in the external validation dataset are shown in Supplemental Fig. 8. The correlation coefficients between MRAC_ZTE_ and CT in 3rd thoracic vertebra (median, 0.82), 7th vertebra (median, 0.81), 11th vertebra (median, 0.84) and 7th rib (median, 0.72) were significantly higher than those between MRAC_Dixon_ and CT (0.24, 0.24, 0.24 and 0.65) (*p* < 0.005), consistent with the findings from the test dataset in institution 1.

## Discussion

In our study, we employed unsupervised deep learning with unpaired training data to generate bone components from ZTE to CT, successfully applying this method for AC of chest PET/MRI. The generated pseudo-CT images accurately depicted bone components with mu values and histograms closely matching those of the patient’s actual CT. Furthermore, integrating these pseudo-CT images into AC for chest PET/MRI enabled the acquisition of quantitative values comparable to PET/CT, demonstrating repeatability within the same patient and reproducibility with external data.

The challenge of AC in PET/MRI was initially addressed in the brain. Techniques such as ultra-short TE (UTE) and ZTE were developed to derive bone components directly from MRI, offering improved quantification compared to the CT atlas method [7, 19]. Subsequently, techniques incorporating machine learning and deep learning were developed to generate more accurate pseudo-CT images [20]. While brain PET/MRI AC has reached clinical equivalence to PET/CT, directly applying these methods to the rest of the body is challenging due to anatomical complexity, motion artifacts including respiratory and cardiac movements, and MR signal intensities similar to those of bone, such as from the lungs or digestive tract gases. Approaches utilizing PET emission data, such as the maximum-likelihood reconstruction of attenuation and activity algorithm or that in combination with deep learning, eliminate the need for CT or MRI and are suitable for the brain [21] as well as body PET/MRI [22–24]. However, inaccurate lung segmentation, particularly in the chest region, can result in image reconstruction errors [25]. Lutetium oxyorthosilicate (LSO) crystals can be used to measure attenuation values; however, because of a low signal-to-noise ratio, noise reduction is necessary for clinical application [26]. Although, time-of-flight PET technologies can mitigate some inaccuracies in AC maps, they still fail to prevent underestimation of uptake in bone and areas surrounded by bone [27].

Deep learning-based approaches primarily utilize convolutional neural networks, such as U-Net [28–30], and GANs [31, 32]. The U-Net approach produces reliable results, particularly for the brain, head and neck, and pelvis regions; however, it requires paired training data with minimal misregistration between MRI and CT. A key innovation of our method is its incorporation of MIND into the GAN loss function, which effectively minimizes distortions caused by image transformations and ensures more accurate learning convergence. To the best of our knowledge, our study is the first to propose a method for generating paired data through unpaired and unsupervised learning using GANs while integrating the MIND concept into AC for PET/MRI. In deep learning, overfitting remains a persistent challenge. In the present study, because an unsupervised learning approach was employed, the risk of overfitting is considered to be relatively low. Furthermore, the validity of the generated images (pseudo-CT) and the absence of overfitting were supported by validation using test data from different patients as well as independent test data from an external institution. Although overfitting can be quantitatively assessed using metrics such as the Fréchet Inception Distance (FID), as discussed below, it is difficult to obtain perfectly matched MRI and CT images even in the same patient. Therefore, in the context of the present study, validation of quantitative PET metrics derived from the actually generated images is considered to be a more appropriate evaluation approach.

PET/CT is frequently used as reference standard to assess the quantitative accuracy of PET/MRI. While many studies perform PET/CT either before or after PET/MRI following a single administration of radiopharmaceuticals, the potential impact of physiological changes in uptake over time must be carefully considered. This issue is particularly pronounced in the body, where discrepancies in patient positioning, breathing conditions, uptake phases and differences in bed design between PET/MRI and PET/CT make perfectly voxel-matched clinical data unattainable. Consequently, in our evaluation of quantitative accuracy using PET/CT data from the same patient, we opted for comparisons of regions of interest placed in corresponding areas rather than voxel-wise analysis. While deformable registration could enable voxel-wise comparisons, any observed discrepancy would reflect both pseudo-CT accuracy and registration error. In such a framework, disentangling attenuation correction performance from registration uncertainty would be challenging without an independent validation of registration accuracy. For these reasons, we intentionally selected the thoracic vertebra and the posterior aspect of the rib as a relatively stable anatomical landmark in the chest. While this approach may introduce measurement errors due to potential discrepancies in ROI placement, it represents a necessary compromise given the practical challenges of obtaining ideal reference data in a clinical setting. This strategy prioritizes robustness and reproducibility over anatomical breadth under realistic clinical conditions.

The relatively wide limits of agreement observed in the repeatability analysis (− 33.2% to + 26.7% for bone SUVmean) should be interpreted in the context of known test–retest variability of body FDG PET. Previous multicenter data [33] have shown that substantial fluctuations in SUV metrics may occur even in stable disease, reflecting physiological and acquisition-related variability. Although the reported thresholds were based on SUVpeak, the magnitude of variability observed in the present study falls within the range reported for physiological PET variability. Importantly, a similar range of limits of agreement was also observed for liver SUVmean, which is not directly affected by bone attenuation modeling. This finding further supports the interpretation that the observed variability reflects global physiological and acquisition-related factors rather than instability specific to the proposed attenuation correction method. In clinical test–retest settings, variability attributable to attenuation correction cannot be fully disentangled from physiological fluctuations, respiratory motion, and minor differences in acquisition and measurement conditions. Therefore, the observed limits of agreement likely represent combined physiological and technical variability rather than instability of the attenuation correction method alone. Importantly, the absence of a statistically significant difference between MRAC_ZTE_ and CTAC should not be interpreted as formal equivalence. Instead, the quantitative agreement should be evaluated based on the magnitude of bias and the limits of agreement observed in the Bland–Altman analysis (Table 2).

Our study has several limitations. First, as a retrospective study, we were unable to evaluate the proposed method using prospectively acquired data, data from the other races or data from the rest of the body (i.e., abdomen, pelvis, extremities). However, given the unsupervised nature of the deep learning algorithm, we anticipate it will maintain robustness when applied to prospective data or data from different races or regions. Second, the study was conducted using a single PET/MRI model, and validation on systems from other vendors remains necessary. The differences in MRI-based attenuation correction methods among PET/MRI systems from different manufacturers mainly lie in the type of MRI sequences used, such as ZTE and UTE. Both approaches enable the detection of bone signal by minimizing the echo time, thereby contributing to the generation of bone components in attenuation correction maps. Therefore, it is considered feasible that PET/MRI systems from other manufacturers could also generate attenuation correction maps including bone components by applying unsupervised learning to MRI and CT data acquired from different patients, in a manner similar to the approach used in this study. Third, this study only evaluated physiological uptake and did not assess pathological uptake. In particular, it is necessary to investigate how quantitative values change when the proposed attenuation correction method is applied to bone lesions and to what extent these values differ compared with CTAC. To enable broad clinical application of the present approach, further evaluation in patient populations with lesions including bone involvement is essential and remains a subject for future investigation. Furthermore, we could not evaluate performance across differences in body habitus such as age, sex, or weight. However, because the proposed deep learning method uses unpaired and unsupervised training, we believe it is more robust to morphological variations such as body habitus and bone pathology compared to conventional methods. The present study involves a substantial amount of offline processing, and therefore immediate clinical implementation of the proposed method is currently challenging. However, the accuracy, repeatability, and external validation of the deep learning algorithm were demonstrated in this study. If the aforementioned limitations are addressed in future work, it will be possible to proceed to the stage of implementing the deep learning algorithm on commercial workstation platforms. Such implementation would enable much faster processing and is expected to facilitate clinical application.

In conclusion, unsupervised deep learning using unpaired chest ZTE and CT datasets successfully generated pseudo-CT images of bone structures with a high correlation to CTAC. When integrated into MR-based attenuation correction, this approach enabled stable and quantitatively consistent physiological uptake measurements in chest PET/MRI under the conditions evaluated in this study. These findings support the feasibility of unpaired ZTE-based MRAC in the chest and provide a methodological foundation for future studies investigating lesion-level quantification and broader clinical applications.

## Supplementary Material

Below is the link to the electronic supplementary material.

Supplementary Material 1
