# Supplementary material for: Zero-TE MRI-based attenuation correction for bone components on chest [18F] FDG PET/MRI: accuracy, repeatability, and external validation of an unsupervised deep learning approach using unpaired PET/CT data
Source: Ann Nucl Med. 2026 May 9;40(8):961–70. doi: 10.1007/s12149-026-02213-0 (PMC13388429; doi:10.1007/s12149-026-02213-0)
Supplement: Supplementary file 1 — Supplementary Material 1 [file 12149_2026_2213_MOESM1_ESM.docx]

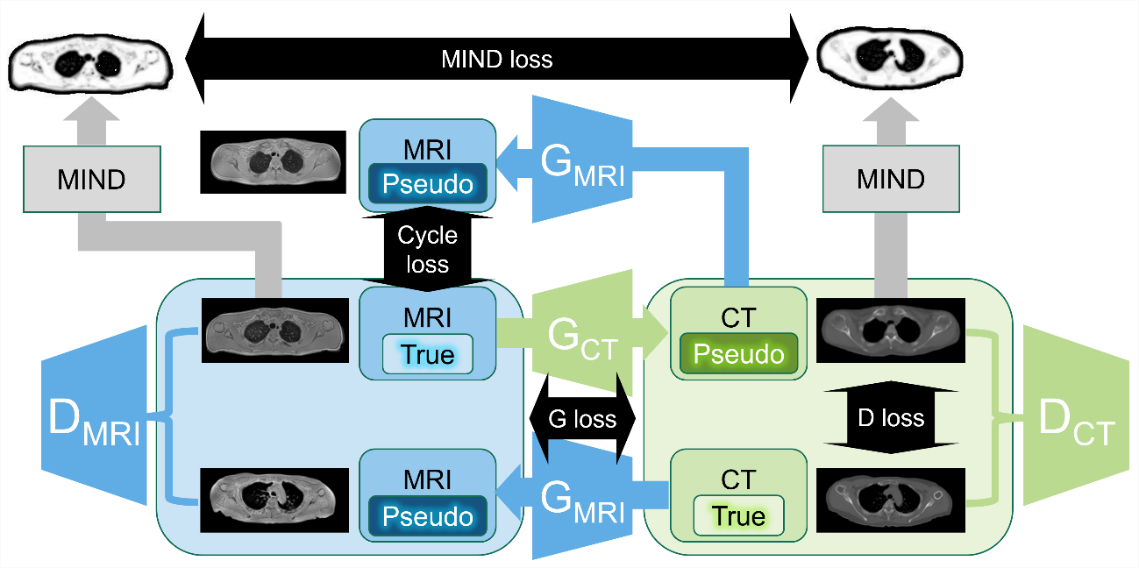
**Supplemental Fig. 1.** Schematic diagram of the deep learning algorithm used in this study. The algorithm utilizes U-GAT-IT, a encoder-decoder approach, for image generation. It integrates an attention module in both the generator and discriminator along with the AdaLIN function, enabling the model to selectively focus on specific regions of the image during the image-to-image translation process. U-GAT-IT learns unpaired ZTE MRI (True) and CT (True) and generates pseudo ZTE MRI (Pseudo) and CT (Pseudo). By adding the MIND loss function to the conventional loss functions, deformation-free pseudo-MRI and pseudo-CT can be generated even when learning unpaired ZTE MRI and CT.

AdaLIN, adaptive layer-instance normalization; CT, computed tomography; MIND, modality independent neighborhood descriptor; MRI, magnetic resonance imaging; ZTE, zero echo-time; U-GAT-IT, unsupervised generative attentional networks with adaptive layer-instance normalization

**
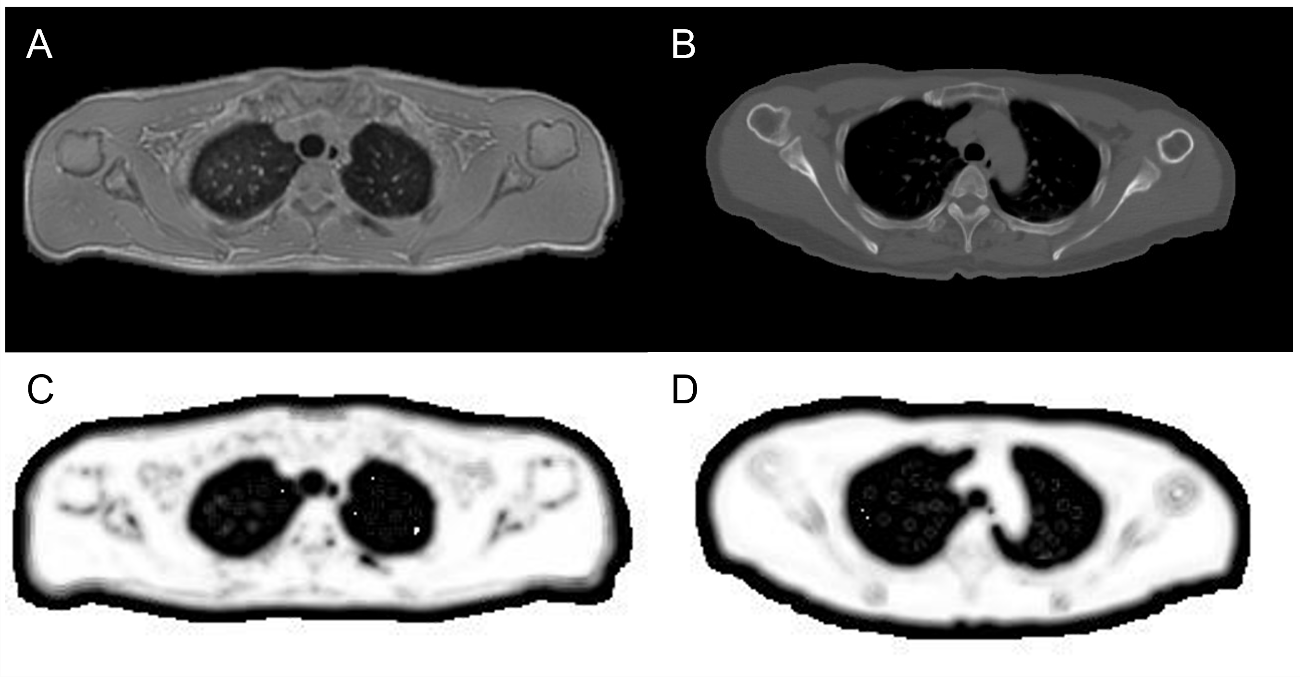
**

**Supplemental Fig. 2.** An example showing the effect of MIND. By extracting local feature structures, numerical descriptors can be generated, and contour information can be preserved between different modalities. The figures show ZTE with center frequency adjustment and background signal removal (A) and CT (B), and their respective images (C) and (D) after processing with MIND.

**
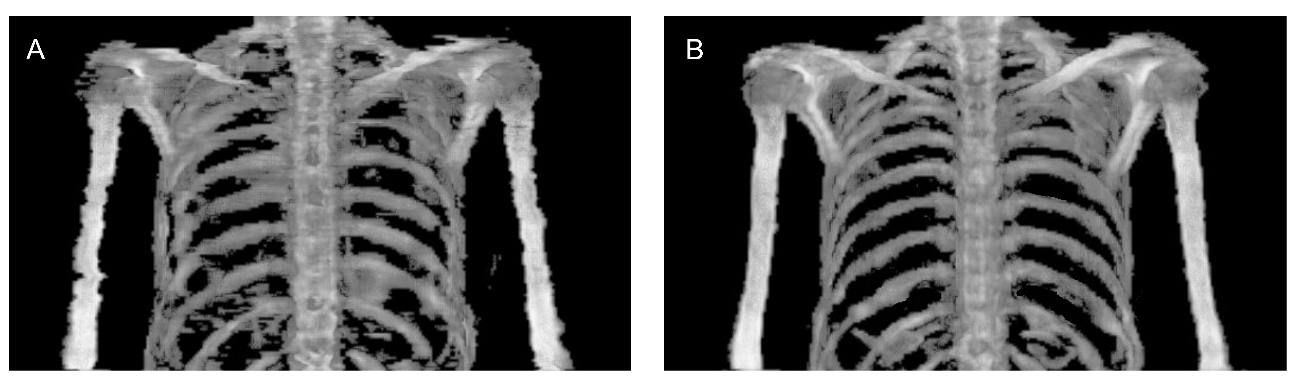
**

**Supplemental Fig. 3.** An example of maximal intensity projection images of bone components of deep learning-based pseudo CT by 2D (A) and 2.5D (B) methods. 2.5D method shows better image quality than 2D method in visualizations of bones especially for the ribs.

**
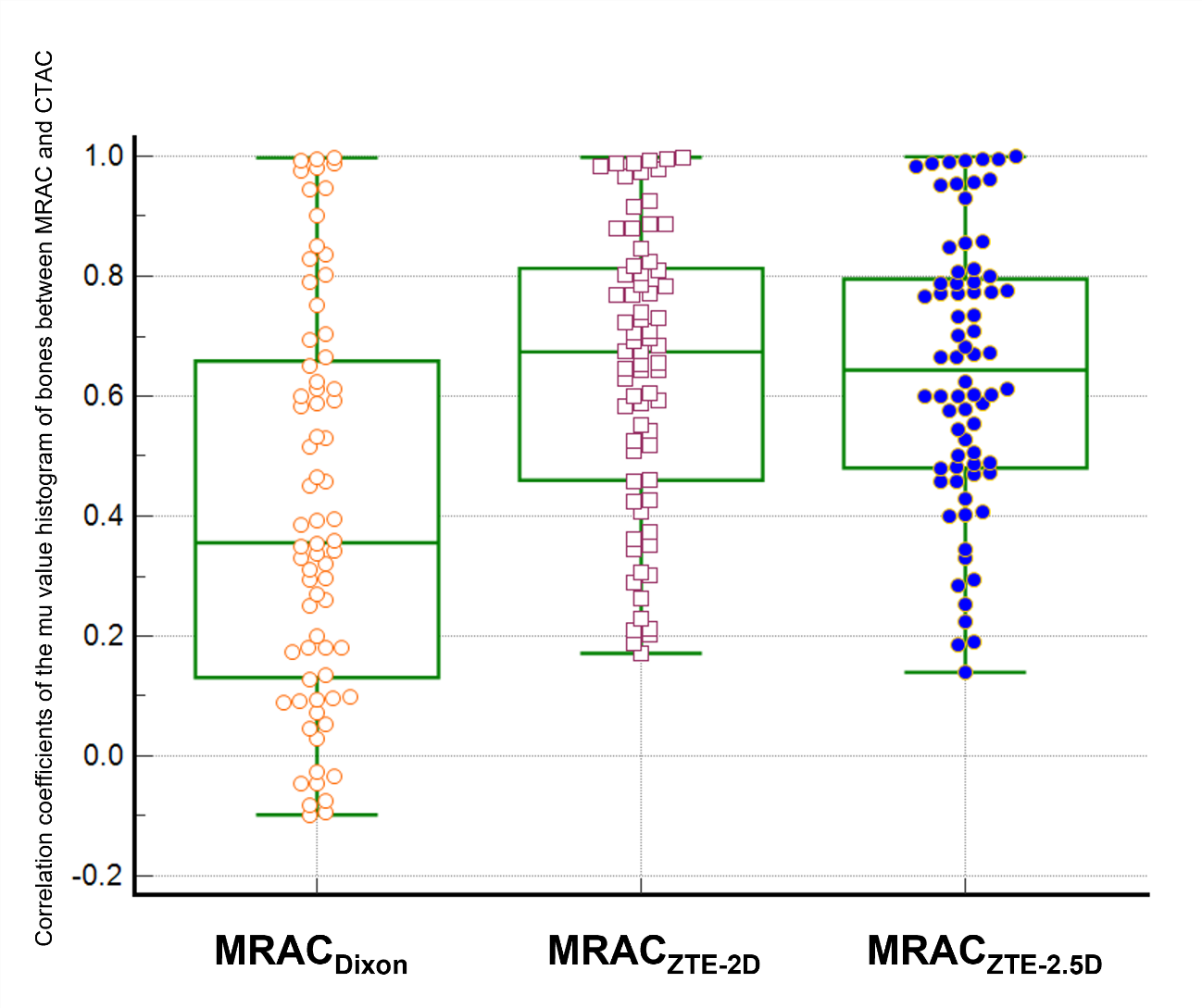
**

**Supplemental Fig. 4.** The mu-map bone histogram comparison between MRAC and CTAC. The correlation coefficients with CTAC was significantly higher in 2D and 2.5D (p<0.0001) with bone method than conventional no-bone MRAC. No significant difference in correlation was observed between the 2D and 2.5D methods.


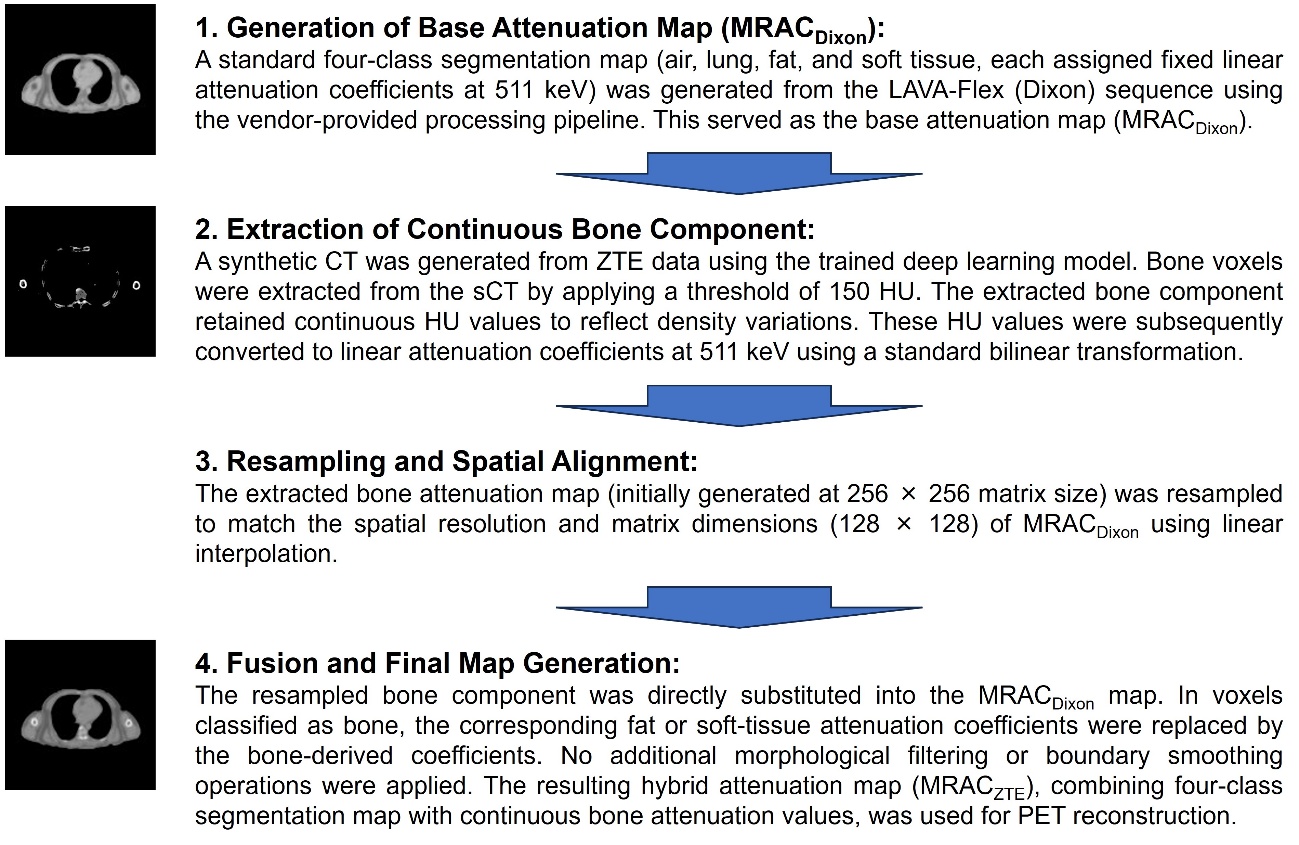


**Supplemental Fig. 5.** Generation of MRAC_ZTE_ via ZTE-Based Bone Fusion A hybrid approach was implemented to generate the final attenuation correction map (MRAC_ZTE_) by integrating a deep learning–derived bone component into the standard Dixon-based MRAC map.

**
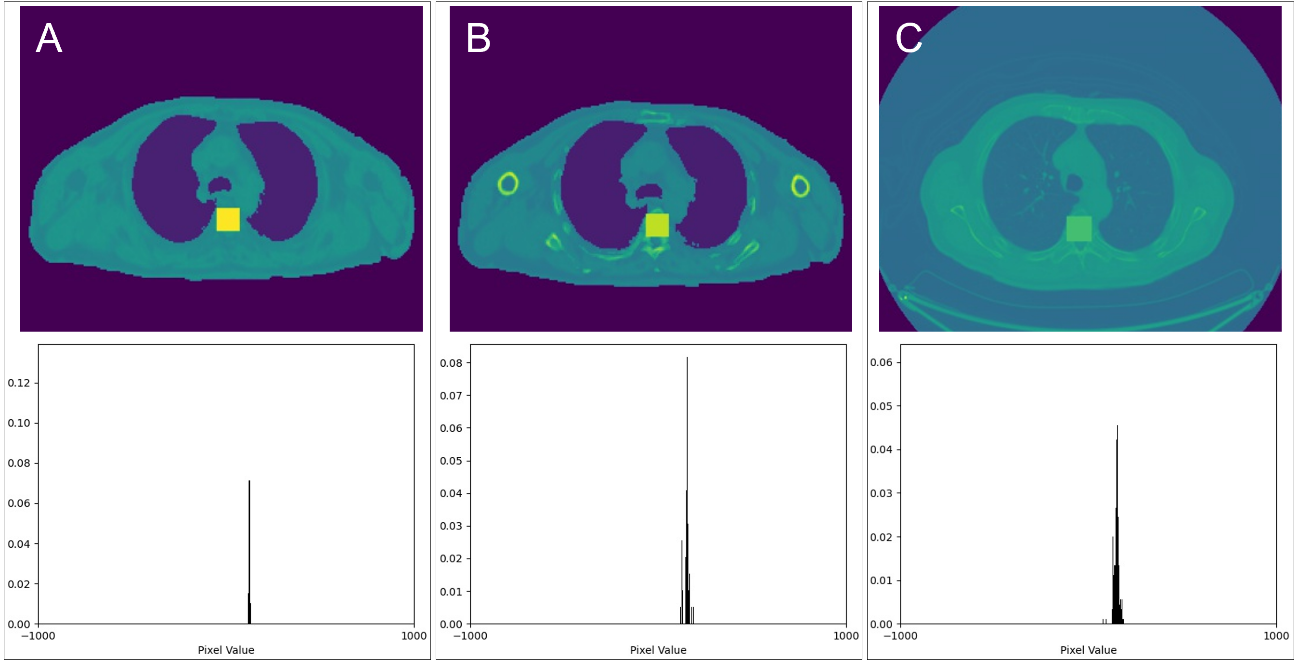
**

**Supplemental Fig. 6.** An example of a histogram comparison between the HU values of a pseudo-CT generated from MRI and those of an actual CT. A 3 cm cubic and fixed region of interest was placed on the body of the seventh thoracic vertebra, and the HU value histograms were compared. It can be observed that the histogram of the pseudo-CT for the 5-class MRAC using deep learning and ZTE (B) is closer to that of the corresponding actual CT (C) than the histogram of the pseudo-CT for the 4-class MRAC based on commercially available product specifications (A).

CT, computed tomography; HU, Hounsfield unit; MRI, magnetic resonance imaging; ZTE, zero echo-time

**
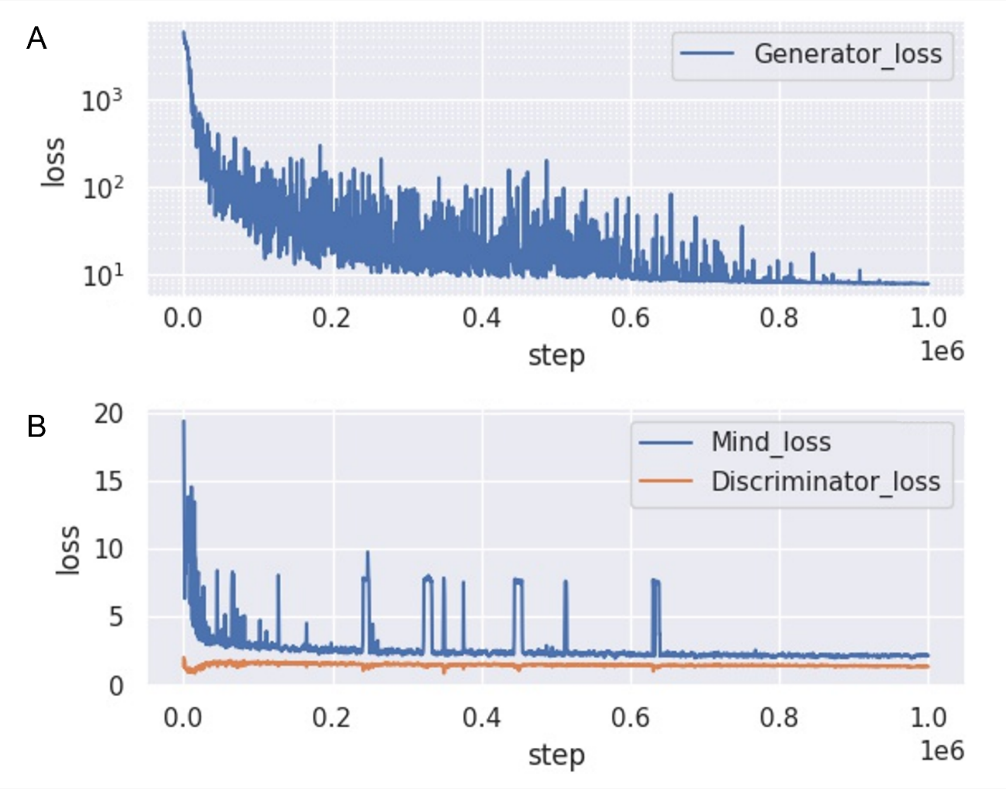
**

**Supplemental Fig. 7.** Graph showing the change over time of the Generator loss (A), MIND loss and Discriminator loss (B) of the Cycle GAN-type deep learning (U-GAT-IT+MIND) used; training was terminated when loss reached a fully reduced and stable phase (0.95×10^6^ steps).

**
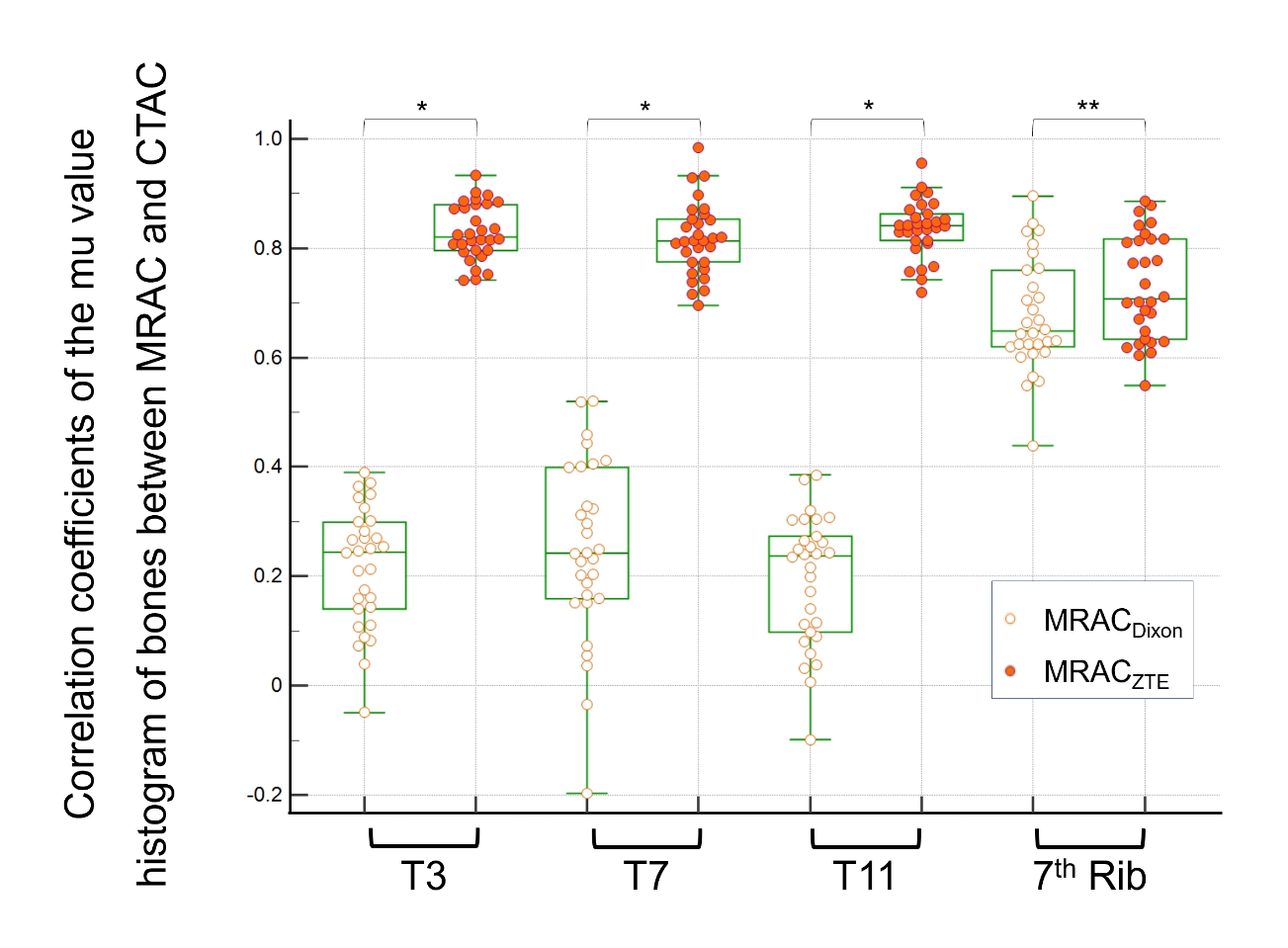
**

**Supplemental Fig. 8.** Box-and-whisker plots showing the correlation between actual CT and pseudo-CT for the bone CT histogram with the region of interest placed on the 3rd, 7th, and 11th thoracic vertebral body and rt. 7th rib in the external validation data (Institute 2). The 5-class MRAC (MRAC_ZTE_) combined with deep learning and ZTE demonstrated a significantly higher correlation with actual CT compared to the commercially available product-specified 4-class MRAC (MRAC_Dixon_) (p<0.005), showing a similar trend as observed at Institute 1.

*, p<0.0001; **, p<0.005

CT, computed tomography; ZTE, zero echo-time

**Supplemental Table 1. PET scan parameters**

| Scanner | Signa PET/MR | Signa PET/MR | Discovery PET/CT 690 |
| --- | --- | --- | --- |
| Applied datasets | Test data A | Repeatability assessment data | Test data B |
| Administrated [^18^F]FDG  Uptake phase | 3.5 MBq/kg  61.1 ± 6.6 min | 3.5 MBq/kg  59.8 ± 5.6 min | 3.5 MBq/kg  60.6 ± 8.2 min |
| Matrix | 192 × 192 | 192 × 192 | 192 × 192 |
| Emission scan duration | 2.5 min | 2.5 min | 2.5 min |
| Reconstruction | TOF-OSEM | TOF-OSEM | TOF-OSEM |
| Subsets | 16 | 16 | 16 |
| Iterations | 2 | 2 | 2 |
| Filter | Gaussian 4mm | Gaussian 4mm | Gaussian 4mm |
| Point Spread Function | On | On | On |
| Respiratory gating | Off | Off | Off |

PET, positron emission tomography; FDG, fluorodeoxyglucose; TOF, time of flight; OSEM, ordered subset expectation maximization.

**Supplemental Table 2. MRI scan parameters**

| Scanner | Signa PET/MR | |
| --- | --- | --- |
| Protocol | Dixon | ZTE |
| Applied datasets | Test data A  Repeatability assessment data | Training data A  Test data A  Repeatability assessment data  External validation data A |
| Sequence | 2-point Dixon | 3D radial |
| Matrix | 256 × 128 | 250 × 250 |
| Scan duration | 18 sec | 5 min |
| FOV | 50 cm | 50 cm |
| TR | 4.9 | 1.4 |
| Slice thickness | 2.6 mm (5.2 mm ZIP2) | 2.0 mm |
| NEX | 1.0 | 4 |
| Imaging mode | 3D | 3D |
| Respiratory gating | Off | Off |

MRI, magnetic resonance imaging; FOV, field of view; TR, time of repetition; ZIP, Zero-fill interpolation processing; NEX, number of excitations; 3D, three dimensional; FSE, fast spin echo; T2WI, T2-weighted image

**Supplemental Table 3. CT scan parameters**

| Scanner | Discovery PET/CT 690 | SOMATOM Force |
| --- | --- | --- |
| Applied datasets | Training data B  Test data B | External validation data B |
| Tube voltage | 120 kV | 100 kV |
| Tube current | 20 mA | 200-600 mA |
| Slice thickness | 3.27 mm | 3.00 mm |
| Reconstruction Kernel  Reconstruction diameter | Soft (mediastinal)  70 cm | Soft (mediastinal)  50 cm |
| Matrix | 512 × 512 | 512 × 512 |
| Respiratory state | Free-breathing | Inspiratory breath holding |

**Supplemental Table 4. Patients’ demographics**

| Demographics | Training data A | Training data B | Test data A/B | Repeatability assessment data | External validation data A | External validation data B |
| --- | --- | --- | --- | --- | --- | --- |
| Patients | 360 | 500 | 25 | 15 | 30 | 30 |
| Sex (M/ F) | 172/ 188 | 239/ 261 | 12/ 13 | 9/ 6 | 14/ 16 | 14/ 16 |
| Age | 66.0 ± 13.6 | 64.7 ± 14.2 | 62.5 ± 18.2 | 66.9 ± 14.1 | 67.1 ± 13.7 | 65.6 ± 13.7 |
| Height [cm] | 159.3 ± 9.7 | 160.2 ± 9.5 | 161.8 ± 8.2 | 159.6 ± 8.8 | 159.6 ± 9.5 | 161.3 ± 8.8 |
| Weight [kg] | 57.5 ± 35.5 | 58.2 ± 34.8 | 59.7 ± 19.9 | 54.2 ± 10.6 | 53.4 ± 9.4 | 55.1 ± 9.2 |

M, male; F, female; Values are mean ± standard deviation

No statistically significant difference between groups (P>0.05)

**Supplemental Table 5. Five-point image-quality scores for DL-based pseudo CT (bone component) by 2D and 2.5D methods (1=poor, 2=fair, 3=good, 4=very good, 5=excellent; the scores were made by three independent readers in consensus)**

| Assessed findings | MRAC_ZTE-2D_ | MRAC_ZTE-2.5D_ | p value* |
| --- | --- | --- | --- |
| Bone delineation | 2.7±1.0 | 3.5±0.8 | <0.0001 |
| Bone continuity | 2.4±0.9 | 3.9±0.5 | <0.0001 |

* Wilcoxon signed-rank test

**Supplemental Table 6. Intraclass correlation coefficient (ICC) of the measured mean HU values on pseudo-CT and actual CT between two readers**

| VOI location | MRAC_Dixon_ (n=85) | MRAC_ZTE_ (n=85) | CT (n=55) |
| --- | --- | --- | --- |
| Subcutaneous fat | 0.999 (0.997 to 0.999) | 0.999 (0.997 to 0.999) | 0.986 (0.972 to 0.991) |
| Lung  Soft tissue  Bone | 0.998 (0.992 to 0.999)  0.992 (0.989 to 0.999)  0.998 (0.991 to 0.999) | 0.998 (0.992 to 0.999)  0.991 (0.988 to 0.999)  0.982 (0.956 to 0.996) | 0.986 (0.972 to 0.991)  0.983 (0.965 to 0.993)  0.980 (0.959 to 0.991) |

Numbers in the parenthesis represents 95% confidence interval.
